# Primary osseous leiomyosarcoma of humerus misinterpreted as aneurysmal bone cyst: A case report and literature review

**DOI:** 10.1097/MD.0000000000039762

**Published:** 2024-09-20

**Authors:** Yong Jin Cho, Young Kwon Koh, Sung-Chul Lim

**Affiliations:** a Department of Orthopedic Surgery, College of Medicine, Chosun University, Gwangju, Republic of Korea; b Department of Pediatrics, College of Medicine, Chosun University, Gwangju, Republic of Korea; c Department of Pathology, College of Medicine, Chosun University, Gwangju, Republic of Korea.

**Keywords:** ABC-like changes, humerus, leiomyosarcoma

## Abstract

**Rationale:**

Primary leiomyosarcoma of the bone (LMSB) is a rare aggressive sarcoma with limited treatment options. Histopathologic and immunohistochemical features are similar to their more common uterine and soft tissue counterparts. However, its broader spectrum of histopathologic features and rarity make diagnostic challenges.

**Patient concerns:**

We present a case of LMSB in a 20-year-old female who presented with left shoulder aching pain for 3 months. An osteolytic intramedullary lesion was found in the left proximal humeral epi-metaphysis.

**Diagnoses:**

Initial open biopsy showed a giant cell tumor of bone with aneurysmal bone cyst (ABC)-like changes. However, an open biopsy followed by extended curettage showed LMSB with ABC-like changes.

**Interventions:**

Wide excision of the lesion and bipolar hemiarthroplasty followed by concomitant chemoradiation therapy was conducted. The mass was completely removed without significant problems.

**Outcomes:**

Complete mass excision and symptomatic improvements were achieved, and no subsequent relapses were observed.

**Lessons:**

The authors encountered a rare case of LMSB. Most occurrences are in the lower extremity and trunk, respectively. ABC-like changes in bone tumors can lead to misdiagnosis. In this case, the ABC-like changes developed from the underlying LMSB as a secondary alteration. A careful examination of the underlying bone tumor is crucial to avoid misdiagnosing it as ABC or exhibiting ABC-like changes. Moreover, there has been no case report of LMSB with secondary ABC-like changes in bone.

## 1. Introduction

Since the first report of leiomyosarcoma of bone (LMSB) in 1965,^[1]^ there have been few sporadic reports each year, but the number of cases diagnosed has increased in recent years due to increased awareness.^[2]^ Most often, somatic leiomyosarcoma occurs in the retroperitoneum, soft tissue of the extremities, and blood vessels, but rarely in the bone.^[3]^ The Japanese Musculoskeletal Oncology Group (JMOG) reported the largest multicenter study on the diagnosis and treatment of LMSB, as well as an analysis of clinical outcomes.^[2]^ It occurs more frequently in women (60%), predominantly in the lower extremity (77%) and trunk (21%), and seldom in the upper extremity (2%). Specifically, there were 24 cases (50%) in the femur, 9 cases (18.7%) in the tibia, 7 cases (14.6%) in the pelvis, 2 cases (4.2%) in the talus, and 1 case (2.1%) each in the fibula, calcaneus, thoracic, lumbar, rib, and humerus.^[2]^

LMSB is initially diagnosed as leiomyosarcoma in 67.4% of cases, as well as spindle cell sarcoma, undifferentiated pleomorphic sarcoma (UPS), osteosarcoma, fibrosarcoma, non-ossifying fibroma, giant cell tumor of bone, and synovial sarcoma.^[2]^

LMSB exhibits the same pathologic characteristics as leiomyosarcoma developed in other tissues. Furthermore, several new chemotherapy regimens have recently proven effective against cutaneous leiomyosarcoma, gastrointestinal, and uterine leiomyosarcoma. It was anticipated that these chemotherapy regimens might also benefit LMSB; however, neither neoadjuvant nor adjuvant chemotherapy has been shown to impact patient outcomes. Thus, performing definitive surgery with negative surgical margins, following an accurate and early diagnosis before any metastasis, is crucial for ensuring a favorable prognosis.^[2]^

Aneurysmal bone cyst (ABC) is a benign, blood-filled cystic neoplasm that develops in the bone. Previously, it was believed to occur as a reaction to trauma or as a reactive lesion caused by an underlying vascular event, but in recent years, it has been considered a true neoplasm caused by rearrangement of the ubiquitin-specific peptidase 6 (*USP6*) gene.^[4,5]^

ABC-like changes are regions of hemorrhagic cystic transformation in both benign and malignant bone tumors, previously known as secondary ABCs. These changes occur due to the disruption of osseous circulation, which is often caused by comorbidities.^[6]^

The authors diagnosed an intramedullary osteolytic lesion in the epi-metaphysis of the humerus of a 20-year-old woman as a giant cell tumor of bone with ABC-like changes on an initial open biopsy. However, extended curettage confirmed this as LMSB with ABC-like changes. Against this backdrop, this study aims to review the rarity and diagnostic pitfalls of giant cell-rich and ABC-like lesions through a literature review, striving to uncover findings that can aid in the rapid and accurate diagnosis of LMSB.

## 2. Case report

A 20-year-old woman presented to the orthopedic outpatient department with a complaint of left shoulder aching pain that started about 3 months before her visit. At her first visit to a private clinic, she was asked to undergo a magnetic resonance imaging (MRI) scan of her left shoulder and was transferred to our hospital with an imaging diagnosis of a suspected giant cell tumor of bone at a general hospital and then a tertiary hospital. At the time of her visit, the patient did not appear excessively ill and had no history of trauma. There was no redness or swelling at the site of the pain.

**Figure 1.**
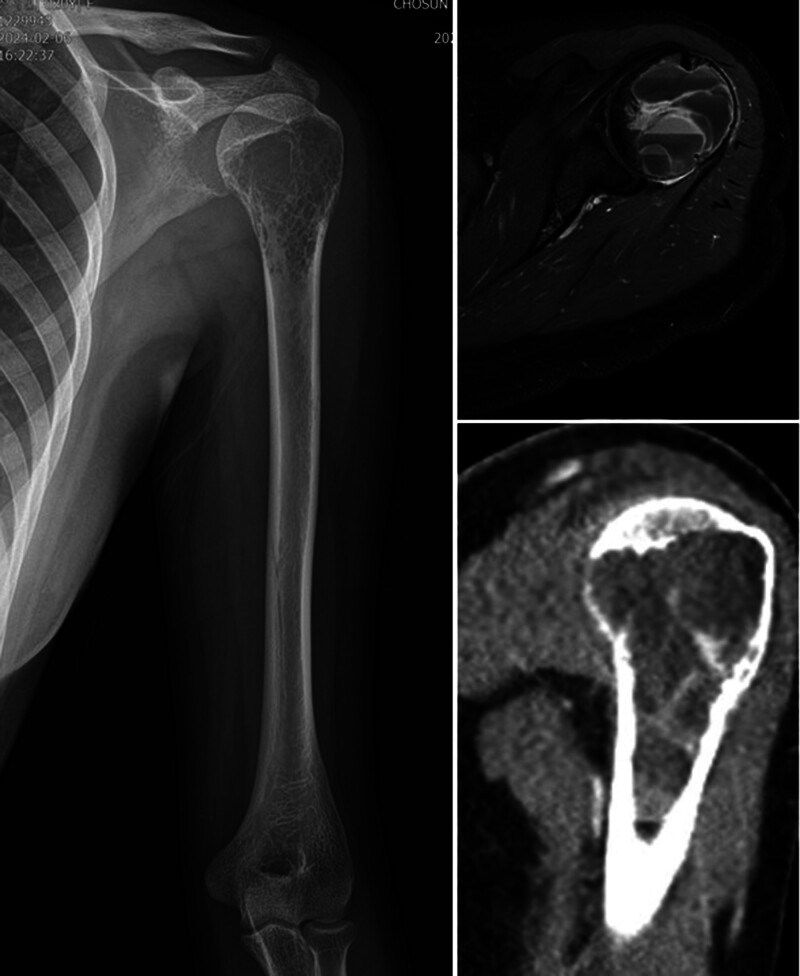
Preoperative images of the left shoulder. A plain radiograph (left) shows an osteolytic intramedullary lesion in the left proximal humeral epi-metaphysis. T2 axial magnetic resonance image shows multiple fluid-fluid levels in the lesion (right upper). The coronal view of computed tomography (CT) image (right lower) shows poorly demarcated multilocular osteolytic intramedullary mass with preserved humeral cortex.

MRI revealed an osteolytic intramedullary lesion of 7.2 × 4.1 cm showing multiple fluid-fluid levels in the left proximal humeral epi-metaphysis, with a relatively preserved humeral cortex. Based on the imaging findings, it was diagnosed as ABC or ABC-like changes, and it seemed necessary to distinguish it from a giant cell tumor of bone (Fig. 1).

The blood tests performed on the day of admission showed 9180/uL (differential count: normal) of white blood cells, 4.83 × 10^12^/L of red blood cells (RBCs), 14.2 g/dL of hemoglobin, 41.9 of hematocrit, and 442 × 10^3^/mL of platelets with no abnormalities.

**Figure 2.**
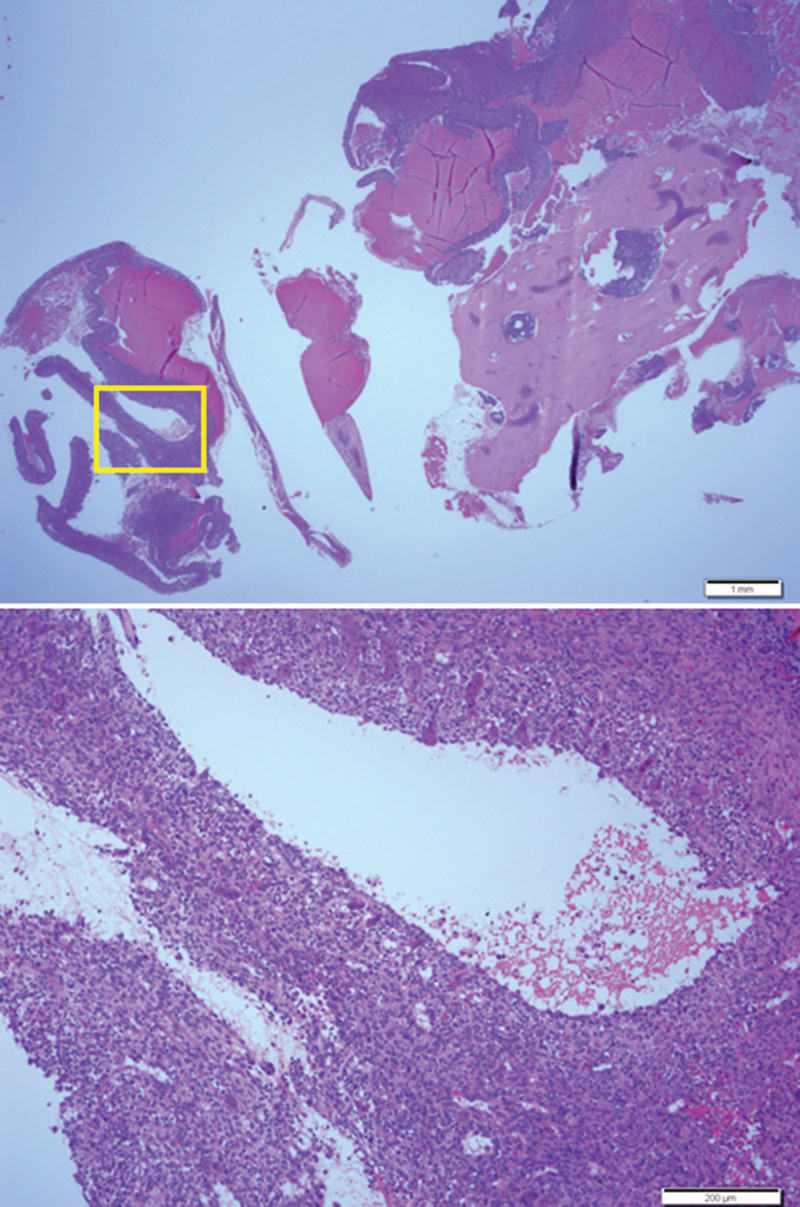
Initial open biopsy shows cavernous or slit-like hemorrhagic spaces which were divided by fibrous septae (top). Higher magnification of the yellow boxed area of the Top shows a fibrous septum comprising fibroblasts, histiocytes, red cells, and multinucleated giant cells (bottom).

After admission to the hospital, an open biopsy revealed that the lesion was composed of multiple fragments of bone and soft tissue. The soft tissue lesion comprised cavernous or slit-like spaces of various shapes and sizes, which were partially or fully filled with RBCs or fibrin, or were empty. These spaces were divided by fibrous septae composed of fibroblasts, histiocytes, red cells, and multinucleated giant cells. The fragments consisted of cortical bone, and their Haversian canals were partially filled with the same cells constituting the fibrous septae. Based on the imaging findings, it was diagnosed as a giant cell tumor of bone with ABC-like changes (Fig. 2).

**Figure 3.**
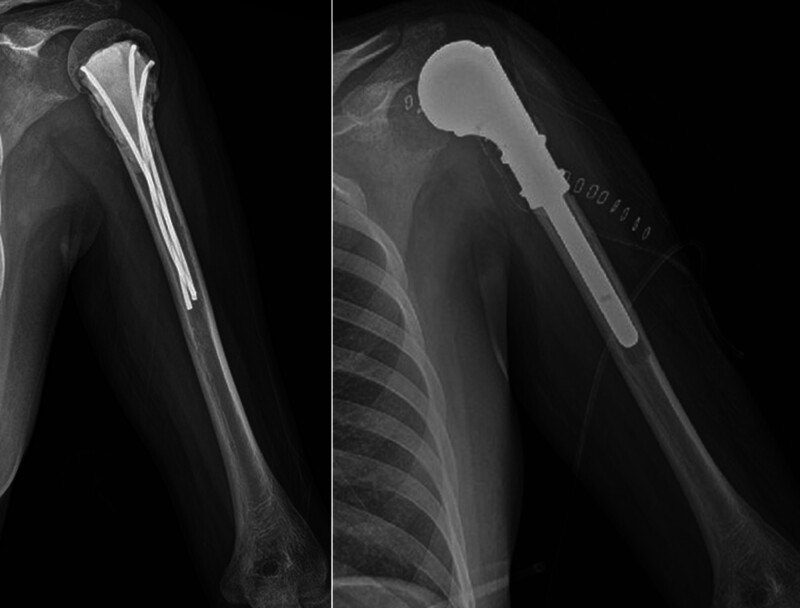
Postoperative images of the left shoulder. Plain radiograph (left) after extended curettage with bone cementing. Plain radiograph (right) after tumor-wide excision and bipolar hemiarthroplasty.

**Figure 4.**
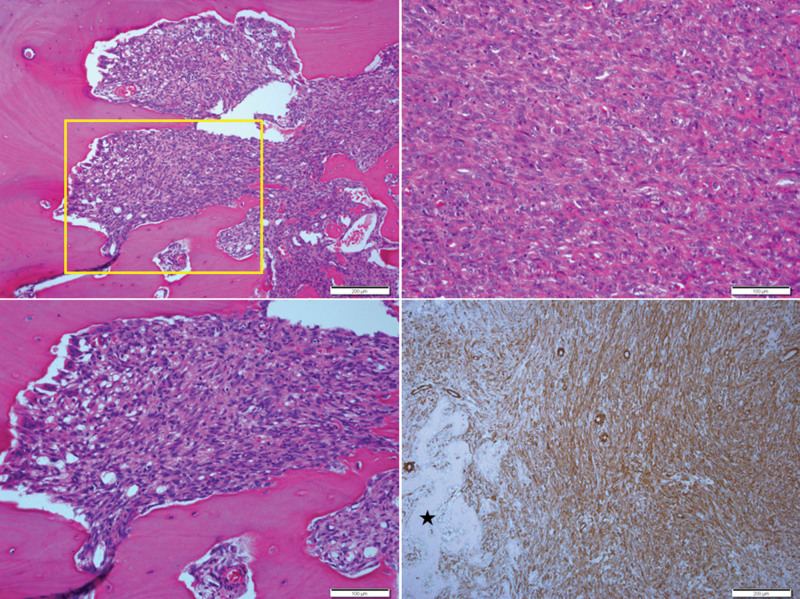
Extended curettage reveals atypical spindle cell proliferation with extensive cortical destruction (upper left). Higher magnification of the boxed area in the left upper illustrates spindle cell sarcoma (lower left). Atypical spindle cells, featuring blunt-ended nuclei, display scattered mitoses (upper right). Tumor cells robustly express smooth muscle actin (SMA), with an asterisk indicating reactive new-bone formation (lower right).

The patient then underwent an extended curettage with bone cementing (Fig. 3 left). The histopathologic examination of the curettage sample revealed extensive atypical spindle cell proliferation, in addition to the findings observed in the initial open biopsy. These atypical spindle cells caused extensive cortical destruction but did not reach the periosteum. Atypical spindle cells had blunt-ended nuclei and showed some scattered mitosis (10 mitoses/10 high-power fields). Tumor cells exhibit marked nuclear pleomorphism and prominent nucleoli, arranged in vaguely intersecting fascicles. The cytoplasm of these cells is eosinophilic and displays indistinct borders. Immunohistochemical analysis demonstrated positive immunoreactivity for smooth muscle actin (SMA), desmin, CDK4, and vimentin, and negative immunoreactivity for Myo-D1, S-100 protein, CD34, MDM2, WT-1, C-Kit, and STAT-6. The Ki-67 index reached 15% in the highlighted areas (Fig. 4).

Large areas of tumor necrosis (approximately 30%) and intratumoral hemorrhage were present. Various degrees of osteoids, observed as large and small sheets or trabeculae, were present at the tumor periphery, causing cortical destruction. This was judged as nonneoplastic new-bone formation.

Based on these findings, it appeared to be LMSB with ABC-like changes, and the Fédération Nationale des Centres de Lutte Contre le Cancer (FNCLCC) grade^[7]^ was 2 (tumor differentiation score: 2, necrosis score: 1, mitotic count score: 2).

The tumor was classified as G2T1M0 and staged as IIA according to the Enneking staging system.^[8]^

With the diagnosis of LMSB, the patient underwent wide excision of the left shoulder joint and bipolar hemiarthroplasty (Figs. 3 (right) and 5). There was no extension of tumor cells into the periosteum or soft tissue around the lesion, with clear surgical resection margins.

**Figure 5.**
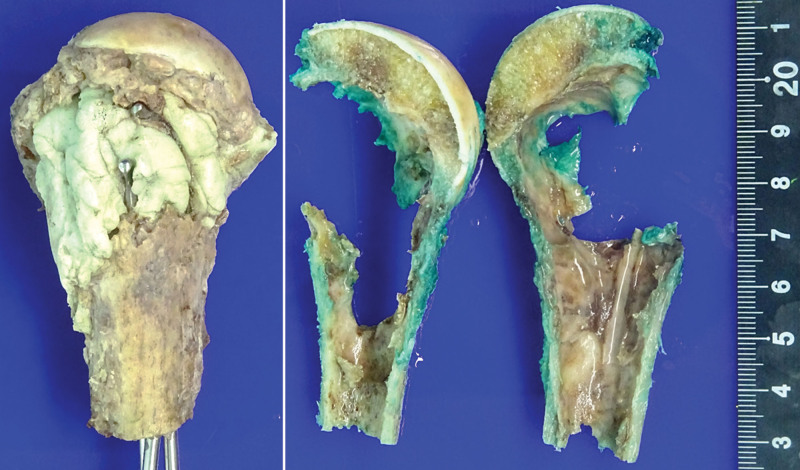
Gross photos following wide excision and bipolar hemiarthroplasty are presented. Extended curettage and bone cementing with a metallic prosthesis are demonstrated (left). A coronal section, after the removal of bone cement and metallic prosthesis, shows a cystic defect with cortical destruction (right).

Positron emission tomography computed tomography (CT) did not reveal any additional bony lesions. Based on these findings, the lesion was diagnosed as primary LMSB with ABC-like changes. Given that the patient was young, only 20 years old, and primary LMSB is exceedingly rare, she was treated with chemotherapy following the COG ARST0332 protocol,^[9]^ which is a widely adopted chemotherapy protocol for soft tissue sarcoma.

As this case had negative surgical margins and no metastases, and the tumor was found to be >5cm in size, adjuvant chemotherapy with doxorubicin and ifosfamide was performed, followed by an additional 55.8 Gy of radiotherapy.

No signs of recurrence have been observed after 12 months of surgery with CT follow-up.

## 3. Discussion

LMSB occurs either de novo or in association with prior irradiation,^[10,11]^ unlike de novo LMSB, 50% of radiation-related LMSB occurs in the craniofacial bones,^[12]^ typically presenting as pain and swelling but occasionally as a palpable mass.^[10]^ Long bones, particularly the distal femur and proximal tibia, are involved in about 70% of cases and the craniofacial bones in about 20%. Imaging studies reveal an osteolytic, radiolucent intramedullary mass accompanied by cortical destruction and soft tissue invasion; 20% to 40% of cases present with a pathologic fracture.^[12–14]^ The median age of patients is 47 years, although ages range widely from 9 to 88 years^.[10,14]^

LMSB exhibits histopathologic features similar to those of its counterparts that occur in the uterus or other soft tissues, consisting of long fascicles of spindle cells permeating through bony trabeculae. There should be no malignant osteoid or chondroid matrix production, and cytologically, smooth muscle differentiation should be evident, with abundant, deeply eosinophilic cytoplasm and elongated, blunt-ended nuclei. The degree of cytologic atypia, cellularity, necrosis, and mitotic activity varies according to histologic grade. The osteoclastic giant cells may be present in varying numbers, from a scattered few to numerous. Some high-grade LMSBs are morphologically poorly differentiated, either lacking or missing morphologic evidence of recognizable smooth muscle differentiation, and resemble UPS. Consequently, immunohistochemical analysis is required for diagnosis.^[10]^ There have been several unique histologic features reported, including an abundance of myxohyaline matrix^[15,16]^ and epithelioid or clear cell morphology.^[17]^ These poorly differentiated tumors can be better identified with immunohistochemical stains. While SMA and muscle-specific actin are almost always positive, desmin is only positive in about 50% of cases, suggesting that it should not be used for screening alone.^[2,18]^

LMSB is focally positive for cytokeratin in about 30% of cases, particularly in epithelioid variants.^[11,18,19]^ Moreover, there are some reports that the S-100 protein is positive in 5% to 80% of cases,^[20,21]^ making the diagnosis of LMSB difficult.

LMSB is primarily required to be differentiated from UPS and fibroblastic osteosarcoma. Immunohistochemical myogenic markers such as SMA and desmin are important in differentiating LMSB from UPS, while focal malignant osteoid formation and negative myogenic markers help differentiate LMSB from fibroblastic osteosarcoma.^[10]^

Due to its low incidence, there is insufficient data on treatment and prognostic factors. However, surgical resection is considered the first-line treatment when feasible. Although postoperative adjuvant radiotherapy has been attempted, no difference in survival rate has been observed compared to the group undergoing surgery alone.^[17]^ Furthermore, it has a poor response to chemotherapy with minimal overall survival benefit.^[2,22]^

The 5-year overall survival rate for LMSB is 62% to 78%,^[2,12,22]^ with stage I 90%, stage IIA 60%, stage IIB 29%, and stage III/IV 0% reported.^[18]^ The prognosis is known to be good if there are negative surgical margins and no metastases at the time of diagnosis.^[2]^ In contrast, tumor size > 8 cm or radiation-related LMSB is associated with a poorer prognosis than otherwise.^[23–25]^

In this instance, the prognosis is anticipated to be favorable, given the negative surgical margins, the absence of metastases, and a tumor size of <8 cm. Local recurrence rates were comparable across tumor grades, with 29% for high-grade tumors and 33% for low-grade tumors. Notably, all metastases manifested within 1 year of diagnosis, with the lungs being the most common site, followed by the axial skeleton and liver.^[18]^

ABC is a benign blood-filled cystic neoplasm of bone, a rare tumor that accounts for 2.5% of all bone tumors.^[26]^ The condition is equally prevalent in both men and women,^[22]^ predominantly affecting patients with still immature skeletal systems, with the highest incidence observed in the first 2 decades of life.^[27]^ ABC-like changes (secondary ABC), on the other hand, represent a reactive process noted in other bone lesions as a secondary manifestation in scenarios such as an underlying vascular event, enhanced venous blood flow, or a response to previous trauma.^[6]^ Secondary ABCs are associated with a variety of primary bone tumors such as giant cell tumor of bone, chondroblastoma, osteoblastoma, Langerhans cell histiocytosis, hemangioma, non-ossifying fibroma, and osteosarcoma.^[28]^ Moreover, fibrous dysplasia and genetic disorder could be associated with secondary ABC.^[29–32]^

In the present case, it was confirmed that LMSB with secondary ABC-like changes. To the best of our knowledge, this is the first case report of LMSB with secondary ABC-like changes in bone. ABC-like changes reflected a reactive process emerging in the occurrence of some events to the underlying LMSB (i.e. minor trauma and physical stresses).

The radiographic characteristics of ABC are distinct and definitive. Conventional radiographs reveal an eccentric radiolucent lesion with expansile bone remodeling. A thin periosteal rim with a multilocular appearance is typically observed. CT imaging displays a well-delineated lytic lesion, encircled by a mostly reactive bone rim. Occasionally, fluid–fluid levels are noted, necessitating further examination via MRI. Although fluid-fluid levels were found frequently in primary osteoblastoma (100%) and chondroblastoma (82%), but highly variable, depending on the primary lesions pathology.^[28]^ This cystic lesion often presents with variable signal intensity, bordered by a rim exhibiting low T1 and T2 signals.^[28,33,34]^

ABC and ABC-like changes are difficult to differentiate because there are no characteristic histologic findings or immunohistochemical markers to distinguish between them. However, since ABC is a true neoplasm that results in *USP6* gene rearrangement, a *USP6* gene rearrangement study is helpful for differentiation.^[4,5]^

Regarding the present case, it is speculated that ABC-like changes were caused by unidentified events during LMSB development due to repeated hemorrhagic cystic change. Furthermore, as ABC-like changes can masquerade as an underlying tumor, thorough sampling and careful examination are required to look for underlying bone tumors, both benign and malignant, that can cause ABC-like changes.

## 4. Conclusion

A lesion located at the epi-metaphysis of the humerus was initially diagnosed as a giant cell tumor of bone with ABC-like changes from an open biopsy. However, extended curettage revealed LMSB with ABC-like changes. A review of the rarity of LMSB, along with the diagnostic pitfalls associated with giant cell-rich and ABC-like lesions, will aid in the swift and accurate diagnosis of LMSB.

## Author contributions

**Data curation:** Yong Jin Cho.

**Funding acquisition:** Sung-Chul Lim.

**Methodology:** Yong Jin Cho, Sung-Chul Lim, Young Kwon Koh.

**Supervision:** Sung-Chul Lim.

**Validation:** Sung-Chul Lim.

**Writing – original draft:** Yong Jin Cho.

**Writing – review & editing:** Sung-Chul Lim.
